# Self-Rated Health as a Predictor of Death after Two Years: The Importance of Physical and Mental Wellbeing Postintensive Care

**DOI:** 10.1155/2017/5192640

**Published:** 2017-08-21

**Authors:** Marie Vejen, Jakob B. Bjorner, Morten H. Bestle, Anne Lindhardt, Jens U. Jensen

**Affiliations:** ^1^Department of Anesthesiology and Intensive Care, Bispebjerg Hospital, Copenhagen, Denmark; ^2^Department of Public Health, Section of Social Medicine, Copenhagen University, Copenhagen, Denmark; ^3^Optum Outcomes, Lincoln, RI, USA; ^4^Department of Anesthesiology and Intensive Care, Nordsjællands Hospital, Hillerød, Denmark; ^5^Department of Respiratory Medicine, Herlev Gentofte Hospital, Hellerup, Denmark; ^6^CHIP & PERSIMUNE, Department of Infectious Diseases, Finsencenteret, Rigshospitalet, Copenhagen, Denmark

## Abstract

**Introduction:**

The objective of this study is, among half-year intensive care survivors, to determine whether self-assessment of health can predict two-year mortality.

**Methods:**

The study is a prospective cohort study based on the Procalcitonin and Survival Study trial. Half-year survivors from this 1200-patient multicenter intensive care trial were sent the SF-36 questionnaire. We used both a simple one-item question and multiple questions summarized as a Physical Component Summary (PCS) and a Mental Component Summary (MCS) score. The responders were followed for vital status 730 days after inclusion. Answers were dichotomized into a low-risk and a high-risk group and hazard ratios (HR) with 95% confidence interval (CI) were calculated by Cox proportional hazard analyses.

**Conclusion:**

We found that self-rated health measured by a single question was a strong independent predictor of two-year all-cause mortality (HR: 1.8; 95% CI: 1.1–3.0). The multi-item component scores of the SF-36 also predicted two-year mortality (PCS: HR: 2.9; 95% CI 1.7–5.0) (MCS: HR: 1.9; 95% CI 1.1–3.4). These results suggest that self-rated health questions could help in identifying patients at excess risk. Randomized controlled trials are needed to test whether our findings represent causality.

## 1. Introduction

The long-term consequences of critical illness are growing in importance since the populations in most countries are getting older and short-term survival is increasing. This raises demand and awareness of postintensive care interventions in patients who initially survived critical illness.

A growing number of studies have investigated mortality after intensive care unit (ICU) treatment and have shown that mortality is high specifically in the first two years after discharge [1, 2] but also that it remains high in the following years [3, 4]. Other studies have focused on predictors of mortality in the ICU setting. Several physical parameters have been suggested to predict survival, such as Acute Physiological and Chronic Health Evaluation Score II (APACHE score II), age, diagnostic group, and severe comorbidity [3]. Critical illness leads to physical, mental, and cognitive sequelae in ICU survivors. These impairments can affect the functional status and exercise ability of the survivors and ultimately the ability to return to life as before their critical illness. It is therefore not surprising that many studies point to lower self-rated health and quality of life in ICU survivors than controls [5–7]. It is now recognized that assessment of outcomes after ICU must include a quality of life assessment [8]. Generic instruments as the SF-36 Health Survey® (SF-36) have been recommended for measuring health-related quality of life. However in an ICU setting such instruments can be labor-intensive and time-consuming, and they have not been routinely included in studies and daily clinic [9]. Although research into rehabilitation for ICU survivors is emerging, uncertainty regarding the best approaches still exists [10–12].

In order to allow for a possible improvement in long-term outcomes it is therefore crucial to identify possible predictors of increased mortality and morbidity following an ICU admission. In this study we aim to determine if two-year mortality in ICU treated patients can be predicted by single-item and multi-item patient-reported measures of health in patients who have survived the first 180 days after ICU admission. Our hypothesis is that self-rated health as well as physical and mental health component scores can be used as predictors of mortality for ICU treated patients. It is beyond the scope of this manuscript to clarify the exact mechanism of causality, but if our hypothesis is confirmed, such findings point to randomized controlled trials testing interventions targeting physical and mental function to improve long-time survival postintensive care.

## 2. Materials and Methods

### 2.1. Study Population

The study was planned and protocolled as a substudy under the Procalcitonin and Survival Study (PASS) trial, a multicenter parallel-group open-label randomized controlled trial (2006–2011). The study was conducted at mixed medical/surgical ICU's in nine regional tertiary care public university hospitals in Denmark [13]. Patients older than 18 years, who were enrolled in the ICU within 24 hours, were considered eligible. Informed consent was given by the patient or next of kin. The study protocol for the trial was approved by the regional ethics committee in Denmark (KF-272-753).

Demographic (age, gender), epidemiologic data (body mass index, preexisting diseases, Charlson Comorbidity Index, and reason for admission), and clinical variables (APACHE II, septic shock, kidney function, and need for mechanical ventilation) were registered in Case Report forms and data were Good Clinical Practice monitored (Good Clinical Practice, GCP, CPMP/ICH/135/95). Vital status was determined 730 days after inclusion in the trial by a search in the Danish death register.

### 2.2. Patient-Reported Outcomes

The SF-36 is a self-completed questionnaire that covers aspects of both mental and physical health with 36 questions. It has been used in multiple studies and it has been validated and found reliable for the use in ICU settings [8, 14]. The questionnaire has been translated into Danish and it has been validated for a Danish population [15]. The SF-36 questionnaire was sent by mail to the patients alive on day 180 after inclusion in the PASS trial with a preaddressed and prepaid return envelope. In the absence of response within 14 days a reminder was sent. We used the first question of the SF-36 as a primary approach to estimate the patients' self-rated health 180 days after ICU treatment. This is a single question pertaining general health status: “In general how would you say your health is?” with response categories Excellent (1), Very good (2), Good (3), Fair (4), and Poor (5). The second SF-36 question “Compared to one year ago, how would you say your health is in general now?,” with response categories Much better (1), Somewhat better (2), About the same (3), Somewhat worse (4), and Much worse (5), was used to evaluate self-rated change in health from before to after ICU treatment (Health transition).

For the self-rated health and health transition questions we stratified the answers into two groups, which the authors decided would make most clinical sense: a low-risk group (answers 1 to 3) and a high-risk group (answers 4 and 5) with the latter representing a poor self-rated health status 180 days after admission to ICU and a worsening in health status since before ICU treatment. The main reason for this dichotomization is that answers “Fair” and “Poor” are semantically different from “Excellent,” “Very good,” and “Good,” the latter representing a positive assessment.

Concerning the remaining questions in the SF-36 questionnaire, the answers were summarized into a Physical Component Summary (PCS) and a Mental Component Summary (MCS) according to the scoring algorithms of the test developers [16, 17]. PCS and MCS were categorized into quartiles, with the low quartile group indicating low scores and poor health.

### 2.3. Statistical Analysis

Data analyses were performed using the SAS System version 9.4 (SAS institute, Cary, NC) and SPSS version 22.0 (SPSS, Chicago, IL). For the descriptive analysis, continuous variables were summarized as medians and interquartile range (IQR) and categorical variables were presented as absolute numbers and percentages. The primary analysis was exploring self-rated health 180 days after ICU treatment and two-year mortality in four Cox proportional hazard multivariable models. Exposure groups (binary) were identified as the high-risk group for the self-rated health and the health transition questions, and the low quartile group for PCS and MCS. The following possible confounders were identified in the literature [2, 3, 18]: age (per year increase), APACHE II score (≥25 versus <25), surgical patient (yes versus no), Charlson Comorbidity Index (≥2 versus <2), Body Mass Index (≥25 versus <25), gender (male versus female), mechanical ventilation (yes versus no), and Estimated Glomerular Filtration Rate (eGFR) (<30 ml/min/1.73 m^2^ versus ≥30 ml/min/1.73 m^2^). These were included in all multivariable Cox models. Differences in mortalities in Kaplan-Meier Plots were assessed by the log-rank test. *p* < 0.05 was considered statistically significant.

### 2.4. Power Calculation

A power calculation for the Cox regression was done before the data analysis phase began for the entire population (*n* = 519). With a power of 0.8 it is possible to detect a hazard ratio of 1.7 (one-sided) or more for a dichotomous predictor, when the significance limit is set to 0.05 and the total event rate is 0.12 (as in this material). The power analysis was performed with Study Size 3.0 (CreoStat HB, Frölunda, Sweden). Since the sample size could not be increased, this power calculation is merely to assist in interpreting the results.

## 3. Results

**Figure 1:**
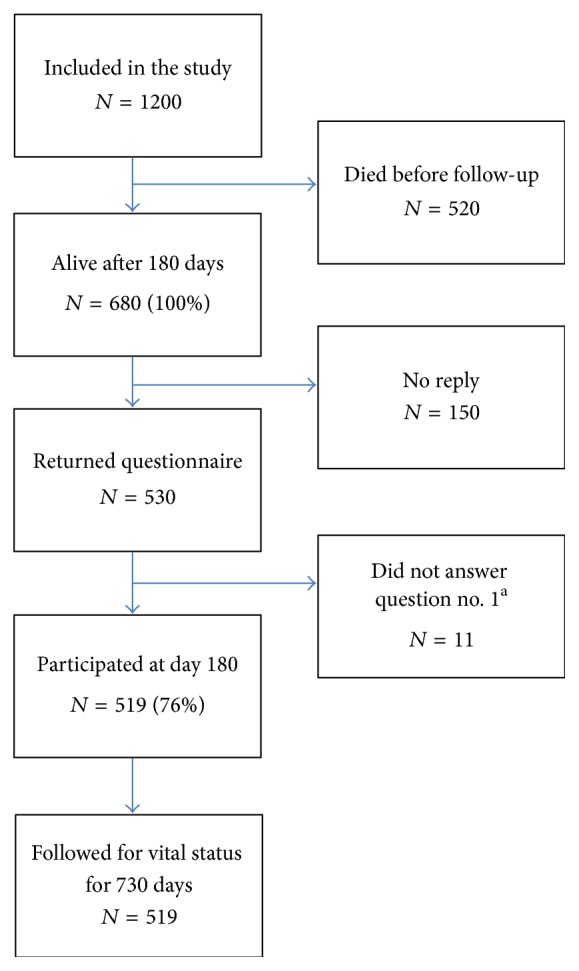
Outline of the study protocol. ^a^Self-rated health question number 1 in SF-36: “In general, would you say your health is” 1–5, Excellent (1)–Poor (5).

In total 1,200 patients were included in the PASS trial. After 180 days 680 were alive, and 530 returned a questionnaire (78%). Eleven patients did not answer the self-rated health and health transition questions, and 519 patients (76%) were therefore included in the data analyses. Follow-up for vital status 730 days after inclusion was 100% (Figure 1).

Four hundred and ninety-four patients (73%) returned a sufficiently complete questionnaire to allow calculations of the component summary scores (MCS and PCS). Baseline characteristics for the population stratified by self-rated health score and for the patients that died before day 180 are shown in Table 1. Not surprisingly, the patients who died before the inclusion day had a tendency towards higher age, higher APACHE II score, longer stay in the ICU, and a higher degree of comorbidities.

### 3.1. Single-Item Question Self-Rated Health Status

In our univariable analysis of self-rated health we found that an answer “Fair” or “Poor” (high-risk group) for the 180 days survivors of ICU treatment was a predictor of two-year mortality (Hazard ratio (HR): 1.9; confidence interval (CI): 1.2–3.1). After adjusting for known or suspected confounders, the high-risk group remained a strong independent predictor of mortality in the period investigated (HR: 1.8; CI: 1.1–3.0). Low eGFR (<30 ml/min/1.73 m^2^) and age were the only variables also showing this quality in both the univariable (eGFR HR: 2.5; CI: 1.1–5.5 and age HR: 1.026; CI: 1.005–1.048) and multivariable analyses (eGFR HR: 2.6; CI: 1.2–6.7 and age HR: 1.029; CI: 1.007–1.052). The Cox regression results for self-rated health are shown in Table 2.

A worsening of the patients self-rated health status over the past year, measured as an answer of “Worse” or “Much worse” to the health transition question, was not a significant predictor of two-year mortality neither in the univariable nor in the multivariable analyses, although the latter showed borderline significance (Table 3).

### 3.2. PCS and MCS

**Figure 2:**
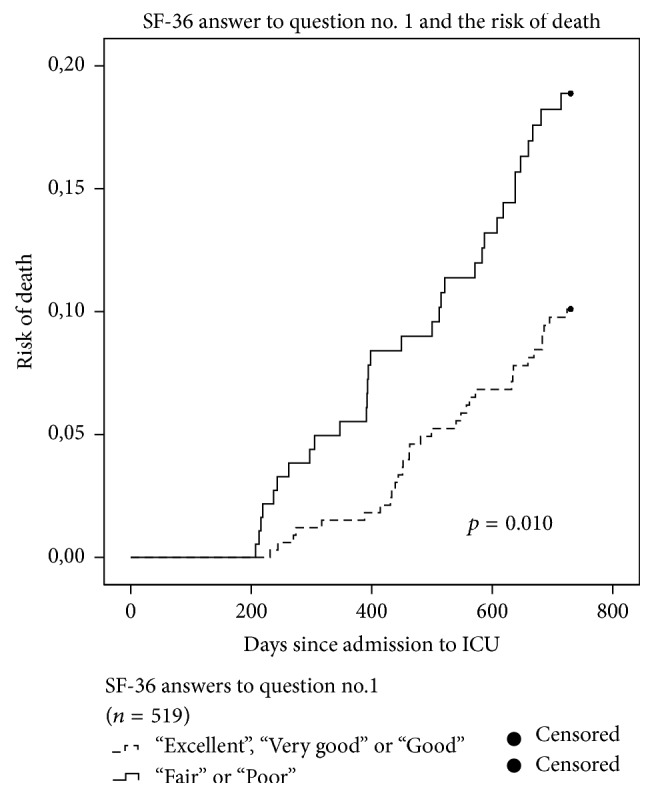
Kaplan-Meier hazard curves for all patients (*n* = 519). Answers to SF-36 self-rated health item: “in general how would you say your health is?” The dashed line represents risk of death for the low-risk-group (answers: “Excellent,” “Very Good,” or “Good”). The fully drawn line is the risk of death for the high-risk- group (Answers: “Fair” or “Poor”).

**Figure 3:**
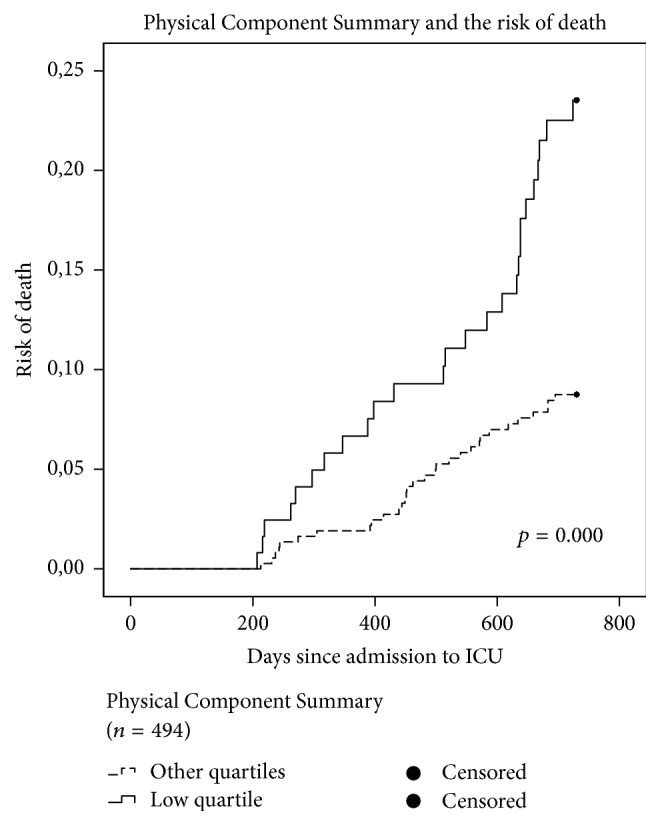
Kaplan-Meier hazard curves for the patients returning a fully completed questionnaire (*n* = 494). The dashed line represents risk of death for the patients with a Physical Component Score in the higher quartiles and the fully drawn line the risk of death for the patients in the lowest quartile group, indicating poor physical health.

A low PCS score was an independent predictor of two-year mortality in both the univariable (HR: 2.7; CI: 1.6–4.5) and multivariable analysis (HR: 2.9; CI: 1.7–5.0) (Table 3). A low MCS score was found to be an independent predictor after adjustment for confounders in the multivariable analysis (HR: 1.9; CI: 1.1–3.4) (Table 3). As in all the other analyses, age and kidney failure remained independent predictors in both analyses. Kaplan-Meier curves for the item on self-rated health (high-risk group versus low-risk group) and for PCS (low quartile group versus other quartiles) are displayed in Figures 2 and 3. There is a significantly higher risk of death in the two groups representing poor self-rated and physical health compared to patients who rate their health better.

## 4. Discussion

In this study of patients who survived minimum 180 days after ICU admission, two patient-reported health measures were strong independent predictors of two-year mortality: a single item about self-rated health and a multi-item scale concerning physical health. The results were robust to adjustment in a multivariable Cox model for other known predictors of mortality in ICU patients. Age and low kidney function were also found to predict mortality independently. A multi-item scale concerning mental health was a statistically significant predictor in the multivariable analyses, but not in the univariable model.

To our knowledge, this study is the first to examine the relation between self-rated health after ICU treatment and two-year mortality. Importantly, the self-rated health items explored in the current study were strong predictors just as other established predictors of mortality in ICU patients, like age and poor kidney function (Tables 2 and 3). The single-item health question was almost as good as predicting mortality as the PCS (Tables 2 and 3). It may seem surprising that people are able to assess their own risk of dying better than other elaborate, expensive, and objective measures used in clinical practice. However, our findings are in concordance with studies of other populations. In a meta-analysis by DeSalvo et al., including 22 community-based cohort studies, study participants' responses to a single-item general health question maintained a strong association with all-cause mortality after adjusting for key covariates such as functional status, depression, and comorbidity [19]. Also studies in disease specific groups like coronary heart disease and cancer have found that self-rated health predicts mortality [20–23]. The association between self-rated health and mortality has been shown to persist over decades [24–26]. Several potential causes of this relationship have been suggested [27]. First, the general health questions may serve as a proxy that summarizes and integrates various components of health status that are not easily measured: preclinical stages of disease, a downward trajectory of health, family disposition for higher mortality, a general susceptibility or resilience, personality traits, life events, social network, and socioeconomic status [20, 28–30]. Second, optimism or pessimism about health may directly affect subsequent health, so that health optimists are more motivated for recovery than people who rate their health as poor.

When people are asked a global question such as the question on self-rated health, they base their response on the information they deem relevant, but we cannot be sure what they have in mind. We therefore included the multi-item scores PCS and MCS, as recommended in previous studies. The multi-item scores have the advantage of being based on a broad profile of items leading to increased reliability at the cost of increased burden and potentially irrelevant questions [31]. The strong relation between the physical health score and mortality may be driven by physical health components that are not well captured by the other measures or by the pathway of an inactive lifestyle discussed above.

The association between MCS of the SF-36 and mortality found in this study is supported by studies in other patient populations. The predictive value of depression has been shown for heart failure patients [32]. Also, Kalantar-Zadeh et al. [33] found that MCS predicts mortality in patients with maintenance hemodialysis. The single-item health transition question could not predict two-year mortality. In our analysis the hazard ratios for general health question 1 and health transition question were fairly similar (1.8 and 1.6 in multivariable analyses). With a power of 0.8 and a given detection limit for HR of 1.7 the results for the health transition question could very well be due to the lack of power.

In spite of the obvious advantages of looking at long-term endpoints in ICU populations, most mortality studies report short-term endpoints like 28-day mortality and composite endpoints sometimes with an even shorter observation time. Variables such as age, comorbidity, admission diagnosis, and severity of illness have been found to predict long-term outcome, although some studies show conflicting results [3]. In our study, apart from self-rated health only a low eGFR and age could predict two-year mortality. Affected kidney function is a known predictor of mortality for patients who have suffered critical illness [34, 35].

### 4.1. Strengths and Limitations

Strengths of this study relate to the high response rate, a study design planned before patient recruitment, the use of validated questionnaire to report self-rated health, and GCP-monitored Case Report forms including data for the multivariate analysis. The follow-up for mortality was 100%. In terms of self-rated health assessment we report a relatively large sample size. Some limitations to our study should also be mentioned. First, as in any other observational study, we may have missed some important confounders. Second, when dichotomizing into a high- and low-risk group there is a risk of selection bias giving the assumingly higher prevalence of preexisting disease. Although we have tried adjusting for comorbidities with the Charlson Comorbidity Index, there is still, as in any cohort study, a risk of residual confounding. Third, the SF-36 questionnaire was sent by conventional mail and we do not know if it was the patients or a next of kin who completed them. However, the use of proxies to assess self-rated health has been validated for the ICU setting in other studies [36–38]. Furthermore we have no data on self-rated health for the patients dying before day 180. This is an inert challenge in the study design and we have therefore included baseline characteristics for the patients dying before day 180 for comparison with the patients enrolled in the study (Figure 1). Finally, while our study represents a relatively large sample size, we are only able to make conclusions for risk variables with a HR above 1.7. Thus, even if the health transition question had a true HR of 1.6, which might be clinically relevant, we are not able to make any conclusions about the predictive value of this question.

Studies have found that rehabilitation programs and exercise for patients with coronary heart disease [39] and chronic obstructive pulmonary disease [40] decrease mortality. Like patients with specific chronic diagnoses, survivors of ICU with impairments need specific rehabilitation and follow-up [4, 10]. To date there are limited numbers of studies testing the effectiveness for physical rehabilitation programs for ICU survivors and the results shows mixed findings [41–43]. Furthermore programs to promote mental recovery after ICU treatment by reviewing signs of depression, anxiety, and posttraumatic stress are emerging, but evidence of the effectiveness is scarce [44].

## 5. Conclusions

In this well characterized ICU population, the findings support the importance of assessing self-rated health. The single-item self-rated health question is easy and convenient to administrate in seriously ill patients, thus a high response rate is possible. We suggest, in line with other studies in different patient populations, that, in an ICU setting, self-rated health measured as a single item could serve as an easy tool that might benefit health care planning and help identifying patients at risk. The PCS and MCS were independent predictors of death. This strongly suggests the need for late physical rehabilitation and interventions specifically directed towards psychological recovery after ICU treatment in order to improve survival. We therefore recommend randomized interventional studies to investigate the effect of specific physical and mental rehabilitation programs in ICU survivors to increase long-term survival and wellbeing of these patients.

## Figures and Tables

**Table 1:** Baseline characteristics. eGFR: Estimated Glomerular Filtration Rate. IQR: Inter Quartile Range. APACHE II: Acute Physiological and Chronic Health Evaluation II. ICU: Intensive Care Unit. SF-36: Short Form 36. NA: no answer.

Variables	Low-risk group^a^ (*n* = 333)		High-risk group^b^ (*n* = 186)		Died before day 180 (*n* = 520)	
	*n*	%	*n*	%	*N*	%
Gender						
Male	183	55	89	48	304	59
Female	150	45	97	52	216	41
Reason for admission						
Medical	205	62	146	79	398	77
Surgical	128	38	40	21	121	23
Charlson Comorbidity Index						
0	127	38	63	34	145	28
1	108	32	56	30	167	32
≥2	98	29	67	36	208	40
Septic shock	111	33	58	31	224	43
Mechanical ventilation	198	60	122	66	392	75
eGFR (ml/min/1.73 m^2^)						
≥60	78	23	42	23	211	41
31–59	95	29	48	26	150	29
≤30	160	48	96	52	159	30

	Median	IQR	Median	IQR	Median	IQR

Age (yr)	65	16	64	16	70	16
APACHE II	17	12	17	10	21	12
Days in ICU	4	7	4	8	8	11
SF-36 Mental Component Summary^*∗*^	53	16	37	17	NA	NA
SF-36 Physical Component Summary^*∗∗*^	45	17	31	10	NA	NA

^*∗*^ *n* = 493, ^*∗∗*^*n* = 494. ^a^Self-rated health, low-risk group: “In general, would you say your health is?” Answers: “Excellent,” “Very Good,” or “Good.” ^b^Self-rated health, high-risk group: “In general, would you say your health is?” Answers “Fair” or “Poor.”

**Table 2:** Cox-regression analyses of predictors of 2-year mortality. ^a^The number of significant digits is different for continuous and categorical data ^b^General health question in the Short Form-36. eGFR: Estimated Glomerular Filtration Rate. IQR: Inter Quartile Range. APACHE II: Acute Physiological and Chronic Health Evaluation II. ICU: Intensive Care Unit. SF-36: Short Form 36.

Risk variable	Univariable hazard ratio (CI 95%)	Univariable *p* value	Multivariable hazard ratio (CI 95%)	Multivariable *p* value
Age (per year increase)^a^	1.026 (1.005–1.048)	0.014	1.029 (1.007–1.052)	0.011

APACHE II score (≥25 versus <25)	0.7 (0.3–1.4)	0.27	0.7 (0.3–1.4)	0.30

Septic shock (Yes versus No)	0.9 (0.5–1.5)	0.60	1.1 (0.6–1.9)	0.73

Charlson Comorbidity Index(2≥ versus <2)	1.6 (1.0–2.7)	0.54	1.5 (0.9–2.6)	0.11

Mechanical ventilation (Yes versus No)	1.2 (0.7–2.0)	0.50	1.2 (0.7–2.1)	0.47

Gender (male versus female)	1.3 (0.8–2.1)	0.34	1.3 (0.8–2.1)	0.35

eGFR (ml/min/1.73 m^2^) (<30 versus ≥30)	2.5 (1.1–5.5)	0.030	2.9 (1.2–6.7)	0.027

Reason for admission (surgical versus medical)	0.9 (0.5–1.5)	0.66	0.9 (0.5–1.6)	0.69

Question: “In general, would you say your health is?”^b^ “Fair/Poor” versus “Excellent/Very good/Good”	1.9 (1.2–3.1)	0.011	1.8 (1.1–3.0)	0.020

**Table 3:** Summary of 4 Cox-regression analyses of self-reported predictors of 2-year mortality. The multivariable analyses adjusted for age, estimated glomerular filtration rate, acute physiological and chronic health evaluation II score, septic shock, Charlson comorbidity index, mechanical ventilation, gender, and reason for admission. See Table 2 for definition of cutpoints. ^a^General health question in SF-36. ^b^Health Transition question in SF-36. PCS: Physical component summary. MCS: Mental Component Summary.

Risk variable	Univariable hazard ratio (CI 95%)	Univariable *p* value	Multivariable hazard ratio (CI 95%)	Multivariable *p* value
Question: “In general, would you say your health is?”^a^ “Fair/Poor” versus “Excellent/Very good/Good”	1.9 (1.2–3.1)	0.011	1.8 (1.1–3.0)	0.020

Question: “Compared to one year ago how would you rate your health in general now?”^b^ “Somewhat worse/Much worse” versus “Somewhat better/Much better/The same”	1.5 (0.9–2.4)	0.13	1.6 (1.0–2.7)	0.057

PCS (low quartile group versus other quartiles)	2.7 (1.6–4.5)	0.000	2.9 (1.7–5.0)	0.000

MCS (low quartile group versus other quartiles)	1.6 (0.9–2.8)	0.09	1.9 (1.1–3.4)	0.027
